# Supplementary material for: Impacts of pre-fire conifer density and wildfire severity on ecosystem structure and function at the forest-tundra ecotone
Source: PLoS One. 2021 Oct 28;16(10):e0258558. doi: 10.1371/journal.pone.0258558 (PMC8553150; doi:10.1371/journal.pone.0258558)
Supplement: S1 Table — Results of linear mixed-effects model of the cumulative sum of carbon content (kg·C·m-2) as a function of depth, depth2, moisture class, and the interaction between depth and moisture class. The model was fit on 205 soil increments from 50 soil monoliths in 15 plots. Random effects of plot and soil monolith nested within plot were used to account for the depth non-independence of soil depth increments sampled within a soil monolith and the spatial non-independence of soil monoliths located within a plot. (DOCX) [file pone.0258558.s001.docx]

**S1 Table. Model results for soil carbon content.**

Results of linear mixed-effects model of the cumulative sum of carbon content (kg·C·m^-2^) as a function of depth, depth^2^, moisture class, and the interaction between depth and moisture class. The model was fit on 205 soil increments from 50 soil monoliths in 15 plots. Random effects of plot and soil monolith nested within plot were used to account for the depth non-independence of soil depth increments sampled within a soil monolith and the spatial non-independence of soil monoliths located within a plot.

| Variable | Estimate ± S.E. | t-value | p-value |
| --- | --- | --- | --- |
| (Intercept) moisture=mesic | 20.7 ± 110.63 | 0.19 | 0.85 |
| Depth | 23.77 ± 25.65 | 0.93 | 0.36 |
| Moisture=mesic-subhygric | -121.66 ± 143.69 | -0.85 | 0.41 |
| Moisture=subhygric | 306.05 ± 154.73 | 1.98 | 0.07 |
| Depth^2^ | 8.00 ± 1.49 | 5.35 | <0.0001 |
| Depth:moisture=mesic-subhygric | 109.21 ± 24.58 | 4.44 | <0.0001 |
| Depth:moisture=subhygric | 23.57 ± 26.08 | 0.90 | 0.37 |
