# Supplementary material for: Impacts of pre-fire conifer density and wildfire severity on ecosystem structure and function at the forest-tundra ecotone
Source: PLoS One. 2021 Oct 28;16(10):e0258558. doi: 10.1371/journal.pone.0258558 (PMC8553150; doi:10.1371/journal.pone.0258558)
Supplement: S2 Table — Number of sites where we observed each species and their average frequency (%) pre-fire, post-fire, and in total. Nomenclature follows Hulten (1968)* and other sources, with current accepted Integrated Taxonomic System nomenclature indicated in square brackets (ITIS 2021)**. (DOCX) [file pone.0258558.s002.docx]

**S2 Table. Understory plant composition.**

Number of sites where we observed each species and their average frequency (%) pre-fire, post-fire, and in total. Nomenclature follows Hulten (1968)* and other sources, with current accepted Integrated Taxonomic System nomenclature indicated in square brackets (ITIS 2021)**

| **Species** | **Code** | **Count of plots where present** | | | **Average frequency (%)** | | |
| --- | --- | --- | --- | --- | --- | --- | --- |
|  |  | **Pre-** | **Post-** | **Total** | **Pre-** | **Post-** | **Total** |
| *Alnus viridis* | alnvir | 24 | 17 | 41 | 0.16 | 0.07 | 0.12 |
| *Andromeda polifolia* | andpol | 13 | 7 | 20 | 0.16 | 0.11 | 0.14 |
| *Anemone parviflora* | anepar | 1 | 1 | 2 | 0.02 | 0.02 | 0.02 |
| *Anemone richardsonii* | aneric | 1 | 0 | 1 | 0 | 0 | 0 |
| *Arctostaphylos rubra [Arctous rubra]* | arcrub | 2 | 2 | 4 | 0.01 | 0.02 | 0.01 |
| *Arctagrostis latifolia* | arclat | 35 | 34 | 69 | 0.33 | 0.22 | 0.28 |
| *Betula nana* | betnan | 46 | 45 | 91 | 0.91 | 0.71 | 0.81 |
| *Betula neoalaskana* | betneo | 11 | 16 | 27 | 0.04 | 0.17 | 0.1 |
| *Betula occidentalis* | betocc | 13 | 17 | 30 | 0.03 | 0.06 | 0.05 |
| *Calamagrostis canadensis* | calcan | 7 | 2 | 9 | 0.08 | 0.03 | 0.06 |
| *Calamagrostis lapponica* | callap | 0 | 37 | 37 | 0 | 0.53 | 0.27 |
| *Calamagrostis stricta* subsp. *inexpansa* | calstr.ine | 1 | 7 | 8 | 0.01 | 0.09 | 0.05 |
| *Carex aquatilis* | caraqu | 1 | 0 | 1 | 0.01 | 0 | 0.01 |
| *Carex bigelowii* | carbig | 44 | 42 | 86 | 0.81 | 0.65 | 0.73 |
| *Carex canescens* | carcan | 0 | 0 | 0 | 0 | 0 | 0 |
| *Carex capillaris* | carcap1 | 1 | 1 | 2 | 0.02 | 0.02 | 0.02 |
| *Carex chordorrhiza* | carcho | 1 | 1 | 2 | 0.01 | 0.01 | 0.01 |
| *Carex concinna* | carcon1 | 0 | 7 | 7 | 0 | 0.07 | 0.03 |
| *Carex gynandra* | cargyn | 1 | 1 | 2 | 0.02 | 0.02 | 0.02 |
| *Carex livida* | carliv | 1 | 0 | 1 | 0.01 | 0 | 0 |
| *Carex membranacea* | carmem | 1 | 1 | 2 | 0 | 0 | 0 |
| *Carex praegracilis* | carpra1 | 0 | 1 | 1 | 0 | 0.01 | 0 |
| *Carex rotundata* | carrot | 2 | 3 | 5 | 0.02 | 0.05 | 0.03 |
| *Carex scirpoidea* | carsci | 1 | 1 | 2 | 0.02 | 0.02 | 0.02 |
| *Carex vaginata* | carvag | 0 | 1 | 1 | 0 | 0.02 | 0.01 |
| *Chamaedaphne calyculata* | chacal | 9 | 7 | 16 | 0.09 | 0.07 | 0.08 |
| *Cornus canadensis* | corcan | 2 | 2 | 4 | 0.03 | 0.02 | 0.02 |
| [***Diphasiastrum complanatum***](https://ecologicalatlas.uaf.edu/index.php/browse-plant-species/atlas-page/?nps_id=702) *[Lycopodium complanatum]* | dipcom | 1 | 0 | 1 | 0.01 | 0 | 0 |
| *Drosera rotundifolia* | drorot | 3 | 4 | 7 | 0.03 | 0.03 | 0.03 |
| *Dryas integrifolia* | dryint.syl | 1 | 1 | 2 | 0.02 | 0.02 | 0.02 |
| *Empetrum nigrum* | empnig | 30 | 9 | 39 | 0.25 | 0.08 | 0.17 |
| *Epilobium angustifolium [Chamerion angustifolium]* | epiang | 0 | 45 | 45 | 0 | 0.69 | 0.34 |
| *Epilobium glaberrimum* | epigla | 0 | 5 | 5 | 0 | 0.02 | 0.01 |
| *Epilobium latifolium [Chamerion latifolium]* | epilat | 0 | 3 | 3 | 0 | 0.01 | 0.01 |
| *Epilobium palustre* | epipal | 1 | 1 | 2 | 0.01 | 0.02 | 0.01 |
| *Equisetum arvense* | equarv | 3 | 4 | 7 | 0.03 | 0.04 | 0.03 |
| *Equisetum fluviatile* | equflu | 1 | 1 | 2 | 0.01 | 0.02 | 0.02 |
| *Equisetum pratense* | equpra | 2 | 1 | 3 | 0 | 0.01 | 0.01 |
| *Equisetum scirpoides* | equsci | 1 | 2 | 3 | 0 | 0.01 | 0.01 |
| *Equisetum sylvaticum* | equsyl | 3 | 3 | 6 | 0.04 | 0.04 | 0.04 |
| *Equisetum variegatum* | equvar | 1 | 0 | 1 | 0.01 | 0 | 0 |
| *Eriophorum angustifolium* | eriang | 2 | 2 | 4 | 0.03 | 0.03 | 0.03 |
| *Eriophorum brachyantherum* | eribra | 1 | 1 | 2 | 0 | 0.01 | 0 |
| *Eriophorum scheuchzeri* | erisch | 1 | 2 | 3 | 0 | 0.02 | 0.01 |
| *Eriophorum vaginatum* | erivag | 42 | 42 | 84 | 0.78 | 0.75 | 0.76 |
| *Festuca altaica* | fesalt | 2 | 0 | 2 | 0.02 | 0 | 0.01 |
| *Galium boreale* | galbor | 1 | 1 | 2 | 0.01 | 0.02 | 0.01 |
| *Geocaulon lividum* | geoliv | 2 | 0 | 2 | 0.02 | 0 | 0.01 |
| *Hedysarum alpinum* | hedalp | 1 | 0 | 1 | 0 | 0 | 0 |
| *Larix laricina* | larlar | 4 | 2 | 6 | 0.02 | 0.03 | 0.02 |
| *Ledum palustre* subsp. *decumbens [Rhododendron tomentosum]* | leddec | 48 | 47 | 95 | 0.93 | 0.78 | 0.86 |
| *Ledum palustre* subsp. *groenlandicum [Rhododendron groenlandicum]* | ledgro | 10 | 7 | 17 | 0.05 | 0.02 | 0.03 |
| *Linnaea borealis* | linbor | 0 | 2 | 2 | 0 | 0.01 | 0 |
| *Luzula multiflora* | luzmul | 0 | 7 | 7 | 0 | 0.05 | 0.03 |
| *Luzula rufescens* | luzruf | 0 | 2 | 2 | 0 | 0.01 | 0 |
| *Lycopodium annotinum* | lycann | 2 | 1 | 3 | 0.02 | 0 | 0.01 |
| *Moehringia lateriflora* | moelat | 1 | 0 | 1 | 0 | 0 | 0 |
| *Myrica gale* | myrgal | 1 | 1 | 2 | 0.01 | 0.02 | 0.01 |
| *Orthilia secunda* | ortsec | 3 | 1 | 4 | 0.01 | 0.01 | 0.01 |
| *Oxycoccus microcarpos [Vaccinium oxycoccos]* | oxymic | 11 | 11 | 22 | 0.11 | 0.11 | 0.11 |
| *Parrya nudicaulis* subsp. *Interior [Parrya nudicaulis]* | parnud | 1 | 0 | 1 | 0 | 0 | 0 |
| *Pedicularis capitata* | pedcap | 0 | 1 | 1 | 0 | 0.01 | 0.01 |
| *Pedicularis sudetica*subsp.*interior* | pedint | 2 | 0 | 2 | 0 | 0 | 0 |
| *Pedicularis labradorica* | pedlab | 8 | 3 | 11 | 0.03 | 0.01 | 0.02 |
| *Pedicularis langsdorffii* | pedlan1 | 1 | 2 | 3 | 0 | 0.01 | 0 |
| *Pedicularis oederi* | pedoed | 0 | 1 | 1 | 0 | 0.01 | 0 |
| *Petasites frigidus* | petfri | 37 | 31 | 68 | 0.43 | 0.27 | 0.35 |
| *Picea glauca* | picgla | 27 | 10 | 37 | 0.09 | 0.06 | 0.07 |
| *Picea mariana* | picmar | 25 | 20 | 45 | 0.18 | 0.22 | 0.2 |
| *Pinguicula villosa* | pinvil | 5 | 3 | 8 | 0.05 | 0.04 | 0.04 |
| *Poa arctica* | poaarc | 0 | 1 | 1 | 0 | 0 | 0 |
| *Polygonum alaskanum [Aconogonon alaskanum]* | polala | 2 | 2 | 4 | 0.02 | 0 | 0.01 |
| *Polygonum bistorta [Bistorta officinalis]* | polbis | 9 | 8 | 17 | 0.08 | 0.06 | 0.07 |
| *Polygonum viviparum [Bistorta vivipara]* | polviv | 0 | 1 | 1 | 0 | 0 | 0 |
| *Populus tremuloides* | poptre | 0 | 1 | 1 | 0 | 0.01 | 0.01 |
| *Potentilla fruticose [Dasiphora fruticosa]* | potfru | 1 | 1 | 2 | 0.02 | 0.02 | 0.02 |
| *Ribes triste* | ribtri | 1 | 0 | 1 | 0.01 | 0 | 0 |
| *Rosa acicularis* | rosaci | 3 | 3 | 6 | 0.04 | 0.04 | 0.04 |
| *Rubus arcticus* | rubarc | 1 | 1 | 2 | 0.01 | 0.01 | 0.01 |
| *Rubus chamaemorus* | rubcha | 42 | 41 | 83 | 0.64 | 0.66 | 0.65 |
| *Salix arbusculoides* | salarb | 1 | 0 | 1 | 0 | 0 | 0 |
| *Salix bebbiana* | salbeb | 3 | 3 | 6 | 0 | 0.03 | 0.01 |
| *Salix glauca* | salgla | 26 | 25 | 51 | 0.09 | 0.1 | 0.1 |
| *Salix myrtillifolia* | salmyr | 1 | 0 | 1 | 0 | 0 | 0 |
| *Salix pulchra* | salpul | 46 | 43 | 89 | 0.58 | 0.48 | 0.53 |
| *Salix reticulata* | salret | 1 | 1 | 2 | 0.02 | 0.02 | 0.02 |
| *Salix richardsonii* | salric | 1 | 1 | 2 | 0.01 | 0 | 0 |
| *Sanguisorba officinalis* | sanoff | 1 | 1 | 2 | 0.02 | 0.02 | 0.02 |
| *Saussurea angustifolia* | sauang | 1 | 1 | 2 | 0.01 | 0.02 | 0.02 |
| *Saxifraga hirculus* | saxhir | 0 | 1 | 1 | 0 | 0 | 0 |
| *Selaginella selaginoides* | selsel | 1 | 1 | 2 | 0.02 | 0.02 | 0.02 |
| *Senecio congestus [Tephroseris palustris]* | sencon | 0 | 2 | 2 | 0 | 0.01 | 0.01 |
| *Spiranthes romanzoffiana* | spirom | 3 | 4 | 7 | 0.01 | 0 | 0.01 |
| *Spiraea stevenii* | spiste | 9 | 4 | 13 | 0.03 | 0.02 | 0.03 |
| *Stellaria longipes* | stelon2 | 5 | 12 | 17 | 0.02 | 0.04 | 0.03 |
| *Thalictrum alpinum* | thaalp | 1 | 1 | 2 | 0.02 | 0.02 | 0.02 |
| *Tofieldia coccinea* | tofcoc | 4 | 3 | 7 | 0.03 | 0.02 | 0.02 |
| *Tofieldia pusilla* | tofpus | 2 | 1 | 3 | 0.02 | 0.02 | 0.02 |
| *Trichophorum caespitosum [Trichophorum cespitosum]* | tricae | 0 | 1 | 1 | 0 | 0.01 | 0 |
| *Vaccinium uliginosum* | vaculi | 48 | 46 | 94 | 0.96 | 0.81 | 0.89 |
| *Vaccinium vitis-idaea* | vacvit | 46 | 44 | 90 | 0.94 | 0.72 | 0.83 |
| *Viburnum edule* | vibedu | 0 | 1 | 1 | 0 | 0 | 0 |

*Hultén E. Flora of Alaska and neighboring territories: a manual of the vascular plants. Stanford University Press; 1968.

**Integrated Taxonomic Information System (IT IS). [cited 22 Jun 2021]. Available: https://www.itis.gov/
