# Supplementary material for: Impacts of pre-fire conifer density and wildfire severity on ecosystem structure and function at the forest-tundra ecotone
Source: PLoS One. 2021 Oct 28;16(10):e0258558. doi: 10.1371/journal.pone.0258558 (PMC8553150; doi:10.1371/journal.pone.0258558)
Supplement: S1 File — Detailed methods and results for seed collections and germination trials. (DOCX) [file pone.0258558.s003.docx]

**S1 File: Seed collection and germination trials**

Seeds for the seeding experiment were collected during the July 2016 sampling within a 5 km radius of each grid. Cones were collected from a variety of age classes of both standing dead and live black spruce. Seeds were separated from cones at the Bonanza Creek LTER lab at University Alaska Fairbanks. As black spruce in semi-serotinous, it was necessary to expose cones to temperatures hot enough to melt cone resin to release seed (Zasada *et al.* 1983; Brown and Johnstone 2012b). Cones were placed on cookie sheets and placed in a 40°C oven for 48 hours. Cones were visually assessed for openness and heated another 24 hours until fully open if necessary. Cones were then placed into quart size mason jars and manually shaken, placed into a soil sieve system (sieve numbers: 4, 10, 20) and again shaken. Contents were collected and sorted to isolate seeds. Ten subsamples of 100 seeds were counted, weighed, and used to estimate the total yield of ~31,000 black spruce seeds.

Viability was assessed with three subsamples of 100 seed. Seeds were first stratified by placing on a paper towel moistened with deionized water, covered, placed in a zip lock bag, placed in a paper bag, and refrigerated for two weeks. Afterwards, seeds were placed in a petri dish lined with filter paper, moistened with DI water, and covered. Petri dishes were surveyed for germination and sufficient moisture (enough to just saturate filter paper) daily for three weeks. The initial trial resulted in an average of 17% (± 1.9% SE) viability. Thus, an estimated 250 seeds (0.187 g) were weighed out for application in each seeded quadrat (N = 120; ~30,000 seeds total). An *a posteriori* viability trial was conducted at Northern Arizona University (n = 300) which resulted in average viability of 15% (± 1.1 SE).
